# Editorial: Microbial involvement in biogeochemical cycling and contaminant transformations at land-water ecotones

**DOI:** 10.3389/fmicb.2024.1525521

**Published:** 2024-12-05

**Authors:** Yizhi Sheng, Xiangfeng Zeng, Linduo Zhao, Yongbin Li

**Affiliations:** ^1^Center for Geomicrobiology and Biogeochemistry Research, State Key Laboratory of Biogeology and Environmental Geology, China University of Geosciences, Beijing, China; ^2^Key Laboratory of Pollution Ecology and Environmental Engineering, Institute of Applied Ecology, Chinese Academy of Sciences, Shenyang, Liaoning, China; ^3^Prairie Research Institute-Illinois Sustainable Technology Centre/Illinois State Water Survey, University of Illinois at Urbana Champaign, Champaign, IL, United States; ^4^Key Laboratory of Industrial Ecology and Environmental Engineering (Ministry of Education), School of Environmental Science and Technology, Dalian University of Technology, Dalian, China

**Keywords:** microbial biogeochemistry, microbial ecology, contaminant transformation, land-water ecotones, bioremediation

Microorganisms, including bacteria, archaea, and fungi, play pivotal roles in facilitating energy and matter exchange, promoting mineral weathering, cycling nutrients, and transforming contaminants (Shi et al., 2016; Dong et al., 2022). Land-water ecotones, such as coastal areas, wetlands, riverine zones, and aquifer recharge regions, are characterized by intensive mass and energy exchanges, dynamic redox processes and varying geochemical conditions. These environments serve as critical transitional zones that support complex biogeochemical interactions between terrestrial and aquatic ecosystems. For instance, the complex interactions between organic matter and iron-bearing minerals at redox interfaces across diverse environments can either enhance or inhibit microbial and extracellular enzyme activity, thereby controlling the carbon budget in those transition zones (Dong et al., 2023; Sheng et al., 2022). In wetland transition zones between groundwater and lakes, processes such as dissimilatory nitrate reduction to ammonia are enriched, exhibiting seasonal variations in microbial activity (Chen et al., 2024). In the continental shelf sediments of China, denitrification is the primary pathway for dinitrogen gas production, followed by anaerobic ammonium oxidation (anammox; Sun et al., 2021). In tropical oceans, biological nitrogen fixation is limited by iron-to-nitrogen ratios, with iron primarily sourced from land and mineral dust deposition (Wen et al., 2022). To counteract metal nutrient limitations, microorganisms have evolved strategies such as producing siderophores to acquire essential metals (e.g., Fe, Mo) from minerals for their growth (Sheng et al., 2023a; Zhou et al., 2024). Understanding how microbial communities and biogeochemical processes respond to both natural variations and human-induced disturbances in these environments is crucial.

The 10 published articles in this Research Topic provide valuable insights into two main themes: (1) microbial community distribution responses to environmental variations, and (2) the impacts of anthropogenic activities on microbial processes, along with potential bioremediation and sustainable development strategies.

The assembly of microbial communities demonstrates remarkable adaptability to fluctuating environmental conditions, including changes in temperature, pH, salinity, oxygen levels, nutrient availability, moisture, hydrodynamic disturbances, and contaminant stress (Sheng et al., 2016; Ruff et al., 2023; Chen et al., 2022; Shu and Huang, 2022). Liu et al. investigated bacterial community dynamics in oligosaline lakes, revealing that temporal succession was the primary driver of community assembly, with temperature, pH, and nitrate significantly influencing microbial structure and function across seasons. Shi et al. explored the effects of environmental gradients on microbial diversity and co-occurrence networks at various depths in Hulun lake, providing insights into nitrogen-cycling taxa and their habitat-specific adaptations in grassland lakes. Xian et al. highlighted the significant impact of water masses, identifiable body of water with a common formation history which has physical properties distinct from surrounding water, on the bacterial composition, topological characteristics and assembly process in the Yangtze River Estuary. These findings offer a theoretical foundation for predicting alterations in microbial communities within estuarine ecosystems under the influence of water masses. Brooks and Field studied responses of microbial communities in freshwater iron mats to hydrocarbon exposure and found that hydrocarbon pollution affects diversity, community structure, and resilience in contaminated ecotones. Guo et al. examined the effects of mining-induced soil fissures on microbial communities, showing that varying fissure conditions impacted soil moisture, pH, and nutrient availability, with rare species playing critical roles in maintaining microbial network stability.

Nutrient and contaminant transformation through microbial degradation, redox reactions, and related processes can either intensify or mitigate their ecological effects. As mobile contaminants cross ecosystem boundaries, the role of microbial communities in detoxification and stabilization becomes crucial for sustaining ecological balance (Sheng et al., 2023b). Li et al. examined the hydrocarbon degradation capabilities of a facultative anaerobic bacterium *Shewanella putrefaciens* CN32 under both aerobic (using O_2_ as an electron acceptor) and anaerobic [using Fe (III) as an electron acceptor] conditions, emphasizing its potential for bioremediation in fluctuating redox environments. Zhan et al. identified the zinc resilience of *Chlamydomonas* sp. 1710, with the half-maximal inhibitory concentration (IC50) values of 225.4 mg/L, suggesting its potential in phytoremediation for metal-contaminated waters. Aké et al. identified microbial strains with high Cr (VI) adsorption capacities, offering insights for Cr-laden wastewater treatment or similar contaminated environments. Feng et al. explored the application of phosphate-solubilizing microorganisms (PSMs) to enhance soil fertility and plant resilience in urban areas, offering a sustainable alternative to chemical fertilizers. Wang et al. reviewed microbial biosorption techniques for radioactive nuclides, highlighting microbial approaches as sustainable alternatives for nuclear waste management in geological environments.

Taken together, this Research Topic advances our understanding of microbial metabolism, distribution, and the key underlying drivers of microbial functions in both pristine and contaminated ecotones and similar environments. By integrating these findings, we will gain a better understanding of global nutrient cycles, contaminant bioremediation, and microbial-environment interactions.
